# The Estimation of Genetic Parameters for Chronic Progressive Lymphedema and Body Traits in the Rhenish German Draught Horse

**DOI:** 10.3390/ani14081214

**Published:** 2024-04-18

**Authors:** Johanna Sievers, Ottmar Distl

**Affiliations:** Institute of Animal Breeding and Genetics, University of Veterinary Medicine Hannover (Foundation), 30559 Hannover, Germany; johanna.sievers@tiho-hannover.de

**Keywords:** heritability, animal model, threshold model, genomic relationship matrix, censoring, genetic correlation

## Abstract

**Simple Summary:**

Chronic progressive lymphedema is an incurable disease in draught horse breeds, particularly prevalent in breeds with genealogical relationships with Belgian draught horses. In this follow-up study, we analysed the heritabilities of this condition in Rhenish German draught horses and in addition, evaluated the genetic and phenotypic correlations with body size, skinfold thickness, and hoof characteristics. Furthermore, we studied heritabilities of chronic progressive lymphedema with models assuming censored data due to the progressive course of the disease. We found high heritabilities of chronic progressive lymphedema in the Rhenish German draught horses, even when we regarded all horses of any age. Over all age groups, the heritability of the score for chronic progressive lymphedema across all four limbs was 0.595 ± 0.131 in the threshold animal model based on pedigree data. Using a genomic relationship matrix yielded very similar estimates, but with slightly smaller standard errors. When assuming censored data for horses with an age below 7 and 8 years, heritabilities reached values of 0.774 ± 0.157 and 0.788 ± 0.168, respectively. Particularly, horses with higher cannon bone circumference, larger frames, and higher skinfold thickness appeared to be disposed to chronic progressive lymphedema. Horses with softer hoof horns seemed to be more susceptible to chronic progressive lymphedema than horses with harder hoof horns. This study showed that genetic disposition to chronic progressive lymphedema is the most important issue and therefore, breeding measures should be initiated to reduce the prevalence of severe cases of this incurable disease in Rhenish German draught horses.

**Abstract:**

Chronic progressive lymphedema (CPL) is a prevalent and progressive disease in Rhenish German draught horses. The objective of our follow-up study was to evaluate the heritability of this disease in Rhenish German draught horses using pedigree-based and genomic relationship matrices. We employed linear and threshold animal models. Models included the random animal effect and effects of breeding association, coat colour, sex, and age within sex, and farm-related factors, on CPL scores. In addition, we estimated heritabilities in models assuming censoring for data when horses were below an age of 1–15 years. The heritabilities of CPL scores across all ages were 0.595 ± 0.131 and 0.482 ± 0.105 in the threshold and linear animal model with pedigree-based relationship matrices, respectively. The restriction of data to horses with a minimum age at examination or accounting for censored data in younger animals showed an increase in heritabilities of CPL scores up to 0.788 ± 0.168 (threshold model) and 0.752 ± 0.153 (linear model) at an age of 7–8 years. Analyses including genomic relationship matrices yielded very similar estimates, but with smaller standard errors than pedigree-based analyses. Heritabilities in threshold models for CPL prevalence (CPL-bin-score) and the number of affected limbs (CPL-bin-sum) were 0.176–0.189 ± 0.061–0.064 and 0.375–0.433 ± 0.164–0.170, respectively. We were able to show moderately to highly positive genetic correlations between the CPL score and cannon bone circumference (0.529–0.825), height at withers (0.338–0.555), and skinfold thickness (0.241–0.517). Using the dichotomous trait for the CPL score and the genomic relationship matrix resulted in corresponding estimates of 0.868, 0.793, and 0.784, respectively. This study showed the great importance of additive genetic variation influencing the expression of chronic progressive lymphedema in Rhenish German draught horses. Therefore, further research is warranted to implement breeding programmes in a small breeding population that exploit the potential of additive genetic differences among animals for reducing the prevalence and severity of lesions of this incurable disease.

## 1. Introduction

Chronic progressive lymphedema (CPL) is an incurable disease, progressing throughout the life of horses with swelling, skin thickening and crusting, hyperkeratosis, fibrosis, skin folds, and nodules, often complicated with exudative wounds and ulcerations or even with verrucous lesions, of the distal limbs [1,2,3,4,5,6,7,8,9,10,11,12,13,14,15,16,17,18,19,20,21]. Secondary bacterial, fungal, and recurrent parasitic infections worsen and complicate lesions [8,9,10,13,14,19,21]. Finally, CPL leads to disability of limbs, loss of appetite, and condition deterioration [4,9,10,19,21]. Draught horse breeds, particularly in breeds with genealogical relationships with Belgian draught horses [2,3,4,10,19], are the only breeds that have been reported as susceptible to this disease. Reports on CPL include Ardennes [6]; Belgian [1,4,8,15,16,19,21,22]; Breton [6]; Boulonnais [6]; Cheval de Trait Auxois [21]; Clydesdale [1,4,7]; Comtois [19]; Danish draught [1]; Friesian [23]; German draught such as Black Forest, Mecklenburg, Rhenish German, Schleswig, Saxon-Thuringian, South German [1,2,3,4,5,10,11,12,13,20,24], and Gypsy Cobs; Gypsy Vanners [14]; Shire [1,4,7]; Trait du Nord; and Trait Mulassier Poitevin [18]. Anecdotal studies presumed large genetic effects on CPL based on progeny records of stallions [1,2,3,5]. In addition, breed disposition may be indicative for genetic involvement. Previous studies in Belgium and across all different breeds of German draught horses showed heritabilities in the range between 0.11 and 0.26 with standard errors of 0.05–0.06 [10,11,12,19,22]. The restriction of data to older horses (>3 years) gave higher heritability estimates for Belgian draught horses compared to horses of all ages (0.26 versus 0.11) [22]. However, within-breed heritabilities in German draught horse breeds varied considerably [10]. In Black Forest and South German, heritability estimates for overall prevalence and prevalence per limb were 0.29–0.12 and 0.14–0.17, respectively. In Rhenish German and Schleswig, corresponding estimates were highest with values of 0.98–0.62 and 0.82–0.55, respectively, and in East German draught horses, heritabilities were at 0.25–0.39 [10]. With the exception of South German (*n* = 455), data sets for single breeds were small and included only 77–141 horses. A limitation of genetic studies in German draught horse breeds may be the small number of horses under study, resulting in higher standard errors for genetic parameter estimates [10,11,12]. In South German, standard errors for heritabilities were at 0.07, but increased to 0.12–0.29 (Black Forest), 0.15 (East German draught), 0.24–0.25 (Schleswig), and 0.32 (Rhenish German) [10]. The small population sizes of these horse breeds, ranging from 178 (Schleswig, N_e_ = 58) to 1085 (Black Forest, N_e_ = 337), 1189 (Rhenish German, N_e_ = 435), and 1974 (South German, N_e_ = 571), impose restrictions on sampling of large data sets (https://www.genres.de/fachportale/nutztiere/rote-liste-nutztierrassen, accessed on 30 March 2024).

The Rhenish German draught horse was the most common horse breed in Germany in the first half of the 20th century. The main breeding areas were the Rhineland, Westphalia, and Saxony. The dramatic changes in agriculture and transportation in the 1960s led to an enormous reduction in demand for draught horses and thus to a sharp decline in the population size of draught horse breeds to just a few thousand (https://www.genres.de/fachportale/nutztiere/rote-liste-nutztierrassen, accessed on 30 March 2024).

The Rhenish German draft horse was developed at the end of the 19th century from local populations and imported stallions of other draught horse breeds from England, Denmark, France, and the Netherlands and, from 1870 onwards, mainly from Belgian stallions. The Wickrath state stud in northern Rhine Prussia concentrated on the Belgian stallion type. The studbook was founded in 1892. As late as 1946, more than 26,000 mares were registered in the studbook (https://www.g-e-h.de/rote-liste-menu/rote-liste, accessed on 30 March 2024) [25]. A few photographs of Rhenish German draught horses are shown in Supplementary Material Figure S1.

A multipoint linkage analysis across German draught horse breeds identified four-chromosome-wide significant loci on horse chromosome (ECA) 1, 9, 16, and 17, and three further breed-related loci on ECA 4, 7, and 10 [24]. For three loci (ECA 1, 10, and 17), immune-response-associated genes were located within these loci. A vicious cycle of interconnected and reinforcing-each-other factors were claimed to be the key players in CLP pathogenesis [21]. The main components may be the lymphatic elastic system with a failing elastic network and chronic inflammation leading to an overregulated inflammatory immune response [26,27,28,29] as well as autoimmune responses [13,21,24]. The genetic disposition may be present in any of the components initiating and driving CPL [10,11,12,19,21,22].

The reason why males and stallions develop more severe CPL lesions at younger ages than geldings and females is still not fully understood [10,11,12,19,22]. The progression of CPL in females is slower but continues to higher ages than in males and geldings among Rhenish German [20]. An arrest of an increase in CPL scores occurred at a mean age of 16 years in males, 18 years in geldings, and 20 years in females and when the minimum age was ≥11, ≥14, and ≥16 years, respectively [20].

In addition, CPL scores are influenced by non-genetic effects, which explain a significant proportion of the phenotypic variance of CPL scores in Rhenish German draught horses [20]. Certain horse-farm-related factors trigger the prevalence and severity of lesions [10,11,12,13,20,30]. Keeping horses on pastures, in outside pens on rubber meadows or pastures, and in paddocks reduces the severity of CPL lesions [13,20]. Stable hygiene and cleanness are important for a lower prevalence [10] and less severe CPL lesions [10,12,20]. Restrictive feeding of concentrates and feeding hay with straw in winter instead of hay silage lowered the risk of CPL lesions [10,20,26]. Nevertheless, the effects of farm-related variables did not explain more than 11.6% of the total phenotypic variance for the CPL score [20]. Extending the model to include the random effect of the horse farm increased the explained phenotypic variance by a further 2.9% to 14.5%. Therefore, our objective of this follow-up study was to analyse the heritability of CPL in Rhenish German draught horses using linear and threshold animal models. Furthermore, we used age-structured subsamples and models for censored data to account for the progressive course of CPL. Relationship matrices were based either on pedigree data or on genomic relationship matrices. Models employed included a random animal effect and the fixed effects related to data structure and horse-farm-related factors, which were significant in our previous study in Rhenish German draught horses.

## 2. Materials and Methods

### 2.1. Ethical Approval

This study was approved by the Institutional Review Board of the University of Veterinary Medicine Hannover (Foundation) and the state veterinary offices from the different German Federal States for North Rhine-Westphalia (registration number 81-02.05.40.19.083), Lower Saxony (registration number 33.8-42502-05-19A465), Thuringia (registration number 22-2684-04-TIH-20-101), Brandenburg (registration number 2347-A-19-1-2020), and Saxony (registration number 25-5131/521/20) on 26 November 2019.

### 2.2. Sample Collection

Design and sampling of horses for this study was described in the previous report [20]. In brief, this study was supported by the Westphalian Breeding Association, the Rhenish Breeding Association, and the North Rhine-Westphalian State Stud and the outline of this study was presented to the breeders in a number of meetings and articles in horse breeders’ journals. All breeders participated voluntarily in this study and all breeding horses present on the particular farms were sampled [20]. We collected the data and EDTA blood samples from November 2019 to October 2021. The horses examined were located on studs in Brandenburg, Lower Saxony, Rhineland, Westphalia, and Thuringia. All horses were registered in the herdbook of the respective regional breeding organisation. All horse farms from the respective region are organised in their regional breeding organisation for Rhenish German draught horses. The data set included 493 horses from 93 studs, with 325 females, 57 geldings, and 111 males. For all 493 phenotyped horses, EDTA blood samples were also available. We sampled 268 horses in Westphalia, 106 in Thuringia, 67 in Brandenburg, 43 in Rhineland, and 9 in Lower Saxony. The average age at examination was 7.77 ± 6.37 years, with horses ≥ 1, 3, 5, 7, 9, 11, 13, and 15 years old being 425, 354, 284, 227, 186, 145, 109, and 75, respectively (Supplementary Material Table S1).

### 2.3. Examination of the Horses

In brief, we recorded all data electronically on farms using the handheld RFID reader APR600 (Agrident, Barsinghausen, Germany) including the transponder and the Universal Equine Life Number (UELN), name, date of birth, coat colour, CPL scores, and body traits. Skinfold thickness was measured on the neck using a Cutimeter (Hauptner, Solingen, Germany) and hardness of the hoof horn at the dorsal wall with a hardness tester (Shore D, Zwick-Roell, Ulm, Germany). More details on data recording and classification of CPL signs can be found in our previous report [20]. The distributions of horses by age in years with corresponding CPL scores and prevalences are given in Supplementary Material Table S1. The examination of each horse for CPL included all four limbs, applying an inspection and palpation of the limbs. Means and standard deviations for body and hoof traits by sex are shown in Supplementary Material Table S2.

### 2.4. Statistical Analysis

#### 2.4.1. Defining Phenotypes

The statistical analysis of data was performed using linear and threshold animal models to estimate additive genetic and residual (co-)variances, heritabilities, and additive genetic, residual, and phenotypic correlation for CPL scores and body traits. Dependent variates used were the CPL score per limb on a scale from 1 to 6, the overall CPL score across all four limbs (CPL score), the highest CPL score per horse (CPL-max) on a scale from 1 to 6, the sum of all CPL scores over all four limbs (CPL-sum). Dichotomous CPL scores were defined as follows: CPL score = 1 for unaffected horses and CPL score > 1 (CPL-bin-score), CPL score > 2, CPL score > 3, and CPL score > 4 for affected horses as 0/1-variates and the sum of the respective CPL-bin-score per limb over all four limbs. Overall CPL scores were defined as follows:

CPL score = 1, if all CPL scores per single limb were 1 or only for one limb a CPL score not greater than 2 was present and all other limbs had a CPL score of 1.

CPL score = 2, if the sum of CPL scores per limb > 5 and <9, the maximum CPL score per limb ≤ 2, and at least two limbs had a CPL score of 2.

CPL score = 3, if the sum of CPL scores per limb > 5 and <13, CPL scores per limb ≤ 3, and at least one CPL score per limb = 3.

CPL score = 4, if the sum of CPL scores per limb > 6 and <17, CPL scores per limb ≤ 4, and at least one CPL score per limb = 4.

CPL score = 5, if the sum of CPL scores per limb > 7 and <21, CPL scores per limb ≤ 5, and at least one CPL score per limb = 5.

CPL score = 6, if the sum of CPL scores per limb > 8 and <25, and at least one CPL score per limb = 6.

The scale for CPL scores was changed from 0–5 to 1–6 and is therefore different from our previous report [20]. The reason for this was because zeroes are treated as unknown trait values in some programmes applied. This linear transformation does not affect (co-)variance estimates.

#### 2.4.2. Models Employed for CPL Scores

The model for CPL scores included the fixed effects of breeding association, coat colour, sex, and the linear and quadratic regressions on age at examination within sex- and farm-related variables, which were significant in our previous analyses [20]. We employed forward and backward selection procedures to confirm significance of the fixed effects and covariates in generalised mixed linear models with a multinomial distribution and a logit link function. In addition, all two-way interactions and other non-linear functions for covariates were tested on their possible significance. Coat colour was found to be significant and was thus retained in the model. The final linear or threshold animal model 1 using quasi-quantitative traits (*y_ijklmn_*) or cumulative ordered probabilities of CPL scores (*η_ijklmn_*) or dichotomous CPL-bin-scores was as follows:

*η_ijklmno_* = ordered categories (or *y_ijklmno_*) = *μ* + breeding association*_i_* + coat colour*_j_* + sex*_k_* + b_1_(age × sex)*_l_* + b_2_(age^2^ × sex)*_m_*+ farm_n1-n6_ + a_o_ + e*_ijklmno_*

where *μ* is an unknown constant common to all horses in the linear model or unknown constants for the thresholds to which the observed CPL scores belong; breeding association*_i_* is a fixed effect, with *i* being Brandenburg-Anhalt, Rhenish, Saxon-Thuringian, and Westphalian including Lower Saxony; coat colour*_j_* is a fixed effect, with *j* being chestnut, black, and bay; sex*_k_* is a fixed effect, with *k* being female, gelding, and male; b_1_(age × sex)*_l_* is a linear regression on age in years within the three different sexes; b_2_(age^2^ × sex)*_m_* is a quadratic regression on age in years within the three different sexes; farm_n1-n6_ involves the six independent fixed effects including OUTS (outdoor facilities for horses in summer with 4 levels), BED (bedding type with 8 levels), CLEAN (time interval for cleaning out the stable with 4 levels), ROUW (type of roughage fed in winter months with 5 levels), CONW (concentrate fed in winter months with 2 levels), and HOFT (length of hoof trimming intervals with 5 levels); a_o_ is a random additive effect of the animal, with *o* = 5160; e*_ijklmno_* is an unknown random residual effect. The definition of levels of farm-related variables and number of horses per level are given in Supplementary Material Table S3.

#### 2.4.3. Data-Selection Scenarios

Model 1 was employed for the different CPL scores of all horses sampled and subsets by age groups using univariate models. Herein, we restricted the analyses for a minimum age from 1 to 8 years in order to test for the influence of the age on heritability estimates for CPL scores. Multitrait models were used for CPL scores, when the scores by limbs or front and hind limbs were analysed.

Animal variables including height at withers; body length; chest circumference; thickness of skinfold at the neck region and at the left front and right hind limb; cannon bone circumference; hoof measures including dorsal wall length, heel length, and front angle; and Shore D hardness of the hoof horn at the dorsal wall were analysed in multivariate animal linear and linear-threshold models together with the CPL score as dependent variates (model 1). The multitrait models included body size traits, skinfold thickness, cannon bone circumference at the front and hind limb, and either hoof measurements of the front limb or the hind limb. The farm-related variables were not parameterised in the model for the body traits as in our previous study [20].

#### 2.4.4. Types of Models Employed

Variance components were estimated for dependent variates with model 1 using VCE 6.0.2 [31]. Variances and covariances were estimated with REML (restricted maximum likelihood) using analytical gradients. The pedigree matrix included 5160 horses, resulting in a generation equivalent to 9.80. Linear model estimations were performed using Residual Maximum Likelihood (REML) with VCE 6.0.2 [31].

In the ordinal threshold model, the observed CPL score is considered as the expression of an underlying continuous variable *l*, which is often called “liability”. This “liability” is rendered discrete by *c* + 1 fixed thresholds *t* (*c* = number of trait categories), each of which divides the observed response into two categories. The probability of obtaining an observation (*y_i_*) of an ordered value *j* given the fixed and random effects and *t_min_* < … < *t_j−_*_1_ < *t_max_* is

$$\mathrm{Pr}(y_{i}=j|fixed\;and\;random\;effects)=\Phi (t_{j}-\eta_{i})-\Phi (t_{j-1}-\eta_{i})$$

where Φ is the standard normal cumulative distribution function, *t_j_* and *t_j−_*_1_ the thresholds, and *η* the linear predictor for an observation.

The linear predictor of the threshold model *η_ijklmno_* presents the function of the expected underlying liability of the CPL score of the *ijklmno*-th animal.

In the threshold models, marginal posterior distributions for all unknowns were estimated in a Bayesian framework using Gibbs sampling. Gibbs sampling was performed with TM software [32]. Flat priors were used for the fixed effects and variance components, resulting in equivalent univariate estimators compared to REML. The number of iterations was set to 500,000, whereof the first 50,000 rounds were discarded as burn-in. The thinning rate was 1/5000 samples for inferences. Residual variance was fixed to 1 for all dichotomous traits in threshold models. Convergence was achieved for all models and traits and numerical problems with estimators going out of bounds were not encountered.

In addition, we calculated the inverse relation matrix of A to be used in the threshold model with TM for all phenotyped horses. Genome-wide genotyping data were obtained using the GGP Equine (71,589 SNPs) genotyping array. After quality control for the genotyping rate (missing rate per sample and genotype) and Hardy–Weinberg equilibrium (*p*-Value < 10^−6^), reducing to autosomal SNPs and pruning at a MAF of 0.05 with PLINK 1.9 [33,34], 48,053 SNPs were left. The genotyping rate was >0.99 and all horses passed the quality control. A linkage disequilibrium (LD)-weighted genomic relationship matrix was calculated with LDAK [35] and then inverted using the procedure IML of SAS, version 9.4 (Statistical Analysis System, Cary, NC, USA, 2024). This inverse matrix G^−1^ instead of the pedigree-based relationship matrix was then used for the estimation of (co-)variances with TM under a linear and threshold animal model.

In addition, we applied censoring for CPL scores using TM software. In this approach, we informed the software that an observed CPL score < 4 for horses less than 6 years old and an observed CPL score < 5 for horses less than 15 years old, but older than 6 years, is lower than the real CPL score. Herein, we assumed right-censored data as recording may be terminated before the horse reaches its highest CPL score. Taking into account censoring, we should be able to evaluate how much information may be lost in models without employing censoring. Models assuming right-censored data included the full data set.

#### 2.4.5. Estimation of Heritability and Correlations

Heritabilities (*h*^2^) were calculated with *h*^2^ = *σ_a_*^2^/(*σ_a_*^2^ + *σ_e_*^2^), where *σ_a_*^2^ was the additive genetic variance and *σ_e_*^2^ the residual variance; additive genetic correlations were obtained from $cov(a_{1}a_{2})/(\sigma_{a1}\;\sigma_{a2}$), with $cov\left(a_{1}a_{2}\right)$ being additive genetic covariance between trait 1 and trait 2 and the product of the square roots of the respective additive genetic variances. Residual correlations were calculated from the residual covariance and variance estimators. Phenotypic variances and covariances are the sum of the respective additive genetic and residual variances and covariances, and phenotypic correlations were calculated using these covariances and variances. The genetic correlation between categorical variates estimated in the underlying scale of a threshold model is identical to the estimate using the observed scale in a linear model [36].

## 3. Results

### 3.1. Heritability Estimates for CPL Using a Pedigree-Based Relationship Matrix

We found heritability estimates ranging from 0.323 to 0.490 in the linear model and from 0.448 to 0.637 in the threshold animal model (Table 1). The summation of CPL scores for front and hind limbs as well as CPL scores for front left limbs and the maximum CPL score for front limbs resulted in lower estimates in comparison to the other CPL traits. Heritability for the CPL score across all four limbs was 0.482 ± 0.105 in the linear and 0.595 ± 0.131 in the threshold model. Additive genetic correlations between CPL scores per limb were larger than 0.95; additive genetic correlations between CPL scores per limb and CPL scores were between 0.91 and 0.97 (Supplementary Material Table S4). CPL-sum and CPL-max for front and hind limbs also showed additive genetic correlations between 0.93 and 0.99 (Supplementary Material Table S5).

Analyses of heritabilities for the CPL score, when data were restricted to a minimum age of the horses, resulted in higher estimates in both animal models, the linear and threshold (Table 2). Restriction to ≥3-year-old horses resulted in heritability estimates of 0.587 ± 0.111 (linear) and 0.720 ± 0.150 (threshold). When considering restriction to an age of ≥5 or ≥8 years in the linear model and ≥7 or ≥8 years in the threshold model, heritability estimates were even higher.

When the censoring of data was assumed, analyses of heritabilities of the CPL score showed the highest estimates in the linear model when data of horses aged < 8 years were censored, and in the threshold model when censoring was applied for horses aged < 9 years (Table 3). Censoring of <10-year-old horses lead to a marked decrease in heritability estimates in the linear and threshold model and an increase in the standard errors to 0.183–0.198 (linear) and 0.183–0.249 (threshold).

### 3.2. Estimation of Genetic Parameters for Body Traits and Their Correlations with CPL Using Pedigree-Based Relationship Matrices

Body traits showed heritability estimates in a range from 0.077 to 0.831 (Table 4). High heritabilities with values of 0.508–0.831 were found for height at withers, circumference of the coronary band at the front and hind limb, length of the dorsal wall at the front and hind limb, cannon bone circumference, and front hoof angle at the hind limb. Additive genetic correlations with the CPL score were highest for cannon bone circumference of both front and hind limbs. The next highest values were obtained for length of the heel wall at the front limb and height at withers. Shore D hardness of the hoof horn was negatively genetically correlated with the CPL score at values of −0.314 (front) and −0.191 (hind). Standard errors of additive genetic correlations were between 0.092 and 0.146. Additive genetic, residual, and phenotypic correlations between body and hoof traits using a linear multitrait animal model are given in Supplementary Material Table S6a–c.

### 3.3. Heritability Estimates for CPL Scores Using Genomic Relationship Matrices

Heritability estimates in the threshold model based on the genomic relationship matrix were slightly higher compared to the model using a pedigree-based relationship matrix for all horses (Table 5), for subsamples with the restriction of age (Table 6), and for data sets with censoring of CPL scores (Table 7). When CPL scores were censored for <13-year-old horses, heritabilities markedly decreased and standard errors increased.

### 3.4. Heritability Estimates for Dichotomous CPL Scores Using Pedigree-Based and Genomic Relationship Matrices

CPL scores were transformed to 0/1-traits (CPL-bin-score), with a CPL score of 1 for unaffected and CPL scores > 1 for affected horses. Heritability estimates were 0.113 ± 0.067 (linear) and 0.189 ± 0.064 (threshold) with the pedigree-based relationship matrix and 0.176 ± 0.061 (threshold) with the genomic relationship matrix (Table 8). For the sum of dichotomous CPL scores per limb over all four limbs (CPL-bin-sum), heritabilities were higher than for the CPL-bin-score, particularly, when using the threshold model and the genomic relationship matrix. The age restriction of data lead to slightly higher estimates compared to the analysis with all horses, but did not exceed values of 0.202 in the threshold models. Analyses using genomic relationships provided slightly lower estimates compared to pedigree-based relationships. Additive genetic correlations of CPL-bin-score and CPL-bin-sum with the CPL score were at 0.927 ± 0.071 (CPL-bin-score) and 0.972 ± 0.049 (CPL-bin-sum), respectively, whereas the corresponding phenotypic correlations were 0.782 and 0.700.

Censoring of CPL-bin-scores had very similar effects on the size of the heritability estimates like age restriction (Table 9). Heritability estimates were highest for <3-year-old horses with a value of 0.203 ± 0.062 using pedigree data and a value of 0.193 ± 0.068 using genomic pedigree data.

### 3.5. Estimation of Correlations between Body Traits and CPL as Dichotomous Trait Using Pedigree-Based Relationship Matrices

Additive genetic correlations with the CPL-bin-score were highest for cannon bone circumference of both front and hind limbs (Table 10). The next highest values were obtained for skinfold thickness, height at withers, chest circumference, and body length. Shore D hardness of the hoof horn was negatively genetically correlated with the CPL-bin-score at values of −0.352 (front) and −0.258 (hind). However, standard errors of additive genetic correlations between the CPL-bin-score and those body traits reached values at 0.152–0.286. Correlation estimates between different definitions of CPL-bin-scores according to the severity of CPL lesions and body as well as hoof traits are given in Supplementary Material Table S7a–c. Using the trait CPL-bin-sum, genetic correlations with cannon bone circumference of both front and hind limbs were even higher with values of 0.695 ± 0.191 (front) and 0.738 ± 0.108 (hind), respectively (Supplementary Material Table S8). The body traits skinfold thickness, height at withers, and body length showed slightly higher additive genetic correlations (0.367–0.492) and Shore D hardness of the hoof horn slightly more negative additive genetic correlations (−0.358 to −0.373) with CPL-bin-sum. The standard errors for these additive genetic correlation estimates were between 0.108 and 0.208.

### 3.6. Estimation of Genetic Parameters for Body Traits and Their Correlations with CPL Using Genomic Relationship Matrices

Heritabilities of body and hoof traits estimated with the genomic relationship matrix were similar to the estimates based on the pedigree data (Supplementary Material Table S9). Correlations with body and hoof traits based on the genomic relationship matrix and a threshold model for the CPL score resulted in slightly higher additive genetic correlation estimates. The traits concerned, in particular, were height at withers (0.555 ± 0.202), chest circumference (0.693 ± 0.266), skinfold thickness (0.517 ± 0.202), cannon bone circumferences (front: 0.825 ± 0.116, hind: 0.671 ± 0.141), and length of the heel walls (front: 0.552 ± 0.252, hind: 0.257 ± 0.561) at the front and hind limbs (Supplementary Material Table S9). For CPL-bin-sum (Supplementary Material Table S10) and CPL-bin-score (Supplementary Material Table S11), the additive genetic correlation estimates showed the same tendency as higher estimates for the same traits. Traits included were height at withers (0.661 ± 0.218, 0.793 ± 0.240), chest circumference (0.693 ± 0.290, 0.556 ± 0.435), skinfold thickness (0.609 ± 0.226, 0.784 ± 0.285), cannon bone circumferences (front: 0.729 ± 0.173, 0.868 ± 0.202; hind: 0.687 ± 0.200, 0.867 ± 0.154), and length of the heel walls (front: 0.563 ± 0.264, 0.449 ± 0.587; hind: 0.481 ± 0.534). Additive genetic correlations with Shore D hardness of the hoof horn were consistently negative for all CPL traits analysed with the genomic relationship matrix (−0.310, −0.305 and −0.446, −0.235 with standard errors of 0.137–0.151 and 0.518–0.665).

## 4. Discussion

In this study, we analysed the heritability of CPL scores using linear and threshold animal models and in addition, compared the heritability estimates with models using genomic relationship matrices and threshold models. The present analyses showed high heritability estimates for CPL scores when including all horses of any ages in the linear and threshold models and even higher estimates when employing age restrictions or censoring of data. Heritability estimates in the threshold models increased from 0.595 to 0.662–0.788 in censored data. We may assume that the progressive course of the disease [1,2,3,4,5,6,7,8,9,10,11,12,13,14,15,16,17,18,19,20,21] may be responsible regarding that in younger horses, the lesions are not fully expressed and therefore, heritability estimates increase when younger horses are not regarded in the analyses or censoring is employed in the analysis. Similarly, the higher heritability estimates with lower standard errors for the CPL scores in the hind limbs may be interpreted in the same way, that expression of CPL is faster and can reach more severe scores than in the front limbs. In front limbs, standard errors may be larger because there is more random error variation due to differences in the progression of the expression of CPL within families. The results of our analyses let us propose that censoring may be useful for horses under an age of 8 to 9 years. In the subsamples of older horses, additive genetic variances and heritability estimates decrease because differences in the severity of the lesions due to the course of the progression of CPL decrease and even the selection of the most severely affected horses may take place [20].

We found very high additive genetic correlations for the CPL scores between all four limbs as well as between the CPL scores of the limbs and the CPL score across all four limbs. Therefore, there is no loss of information when using the CPL score across all four limbs instead of the CPL scores for each limb. In addition, we found high phenotypic correlations between limbs and for the CPL score across all limbs with CPL scores of the limbs.

Employing a genomic relationship matrix increased heritability estimates for CPL scores to a slight extent. Due to the deep pedigree matrix, the additional increase was small but evident. However, an exception was the CPL trait defined as the sum of the dichotomous CPL-bin-scores over all four limbs with an increase from h^2^ = 0.375 to h^2^ = 0.433 for the genomic pedigree matrix. As our data included horses of both sexes, and even geldings, from more than 25 birth years with genotypes and phenotypes and as no selection by phenotypes or estimated breeding values was performed, we assume that the overestimation of heritabilities seems very unlikely [37].

On the other hand, distinguishing only between unaffected and affected horses resulted in much smaller heritability estimates with values of 0.11–0.25 in linear and 0.18 to 0.20 in threshold models. This may indicate that a larger part of the additive genetic variation is due to differences in the severity of CPL lesions and a smaller part regarding whether a horse is affected by CPL or healthy. This offers breeders the option that breeding against CPL scores may first lead to milder signs of the CPL lesions and to a slower progression of CPL lesions with age and in the next stage also to horses being less susceptible to CPL at all.

The present results revealed much higher heritability estimates for CPL scores when compared to the previous study for Belgian draught horses [22]. The previous estimates across all German draught horse breeds, based on linear animal models, were 0.211 for the prevalence as the 0/1-trait across all limbs and 0.235 for the number of CPL-affected limbs [10]. These estimates are comparable to the results of the present study for the 0/1-traits in the linear and threshold model for CPL-bin-score, but higher estimates were obtained for the sum of the dichotomous CPL scores over all limbs in the threshold model, particularly with the genomic relationship matrix. It seems that the sum of the dichotomous CPL scores over limbs contains additional information on the degree of the severeness of the CPL lesions as the affection of CPL will not start in all limbs at the same age but progresses from the hind limbs to the front limbs [10,20]. In the present data, on average, more than two limbs were affected with 5 years of age and more than three limbs with the age of 8 years.

The rather high heritability estimates of 0.824–0.979 for the prevalence and 0.427–0.618 for the number of CPL-affected limbs in a small sample of Rhenish German in a previous study by Wallraf [10] may either indicate that at this time there were even greater additive genetic differences in this population studied or non-random sampling may have contributed to these high estimates. Contrasting results for East German draught horses, which genealogically belong to Rhenish German [38], were reported in the same previous study with heritability estimates of 0.248–0.359 (prevalence) and 0.392–0.501 (number of affected limbs) [10]. The small data sets for the Rhenish German from West and East Germany in the previous study [10] seem to have influenced the size of the heritability estimates. Whether horse lines that have been lost contributed to larger additive genetic differences cannot be excluded. The rather high prevalences of CPL for Rhenish German draught horses of 0.961 in West Germany and 0.812 in East Germany in this previous study [10] let us assume that a very low number of horses has caused these rather high heritability estimates with linear models. Nevertheless, the additive genetic variation to be exploited for breeding programmes may be assumed as larger in the present German population than in Belgian draught horses. The reason for this result may be due to the influence of Belgian stallions in the former time, which were suspected to have sired progeny with a high prevalence of CPL [2,3,4,5]. This way, sire lines may have been selected with large differences for CPL susceptibility. The moderate-to-high positive additive genetic correlation of CPL scores and CPL-bin-scores with body size traits may also suggest that incrossings of large-framed Belgian stallions may have increased the risk of CPL and development of more severe CPL lesions. A limitation of the present study may be seen in the number of horses studied, and consequently the impact of families with extreme trait values may have a larger effect on heritability estimates in comparison to studies using large data sets. Furthermore, standard errors for additive genetic correlation estimates were for some body traits with the binary CPL traits rather high. Therefore, additive genetic correlation estimates with rather high standard errors need to be critically scrutinised. Inbreeding coefficients estimated from pedigree data were lowest in Rhenish German (1.80%) and even higher in South German (2.79%) [38]. We propose therefore that positive assortative matings, which can increase inbreeding and inflate additive genetic variance [39,40], may only have a small influence on heritability estimates in Rhenish German. The small population sizes of the Rhenish German draught horse and stud farms with predominantly few horses, which are widely distributed across Germany, are limiting factors for increasing the number of horses studied to several thousand animals.

Body and hoof traits had moderate-to-high heritabilities, particularly, height at withers, circumference of the coronary band, and length of the dorsal wall of the hooves. The present study suggests that breeding for large-framed horses increases the risk for Rhenish German to be affected by CPL and to develop more severe lesions than smaller-framed horses [2,3,30]. Additive genetic correlation estimates with body length and chest circumference have to be considered with caution due to their large standard errors, whereas standard errors for height at withers were much smaller. The residual and phenotypic correlations with the height at withers were smaller than the additive genetic correlations and therefore, selection on phenotypic traits may be less effective than the use of estimated breeding values for CPL scores and height at withers. Rather high additive genetic correlations were found for cannon bone circumference in the front and hind limb with CPL scores and even higher ones for CPL prevalence and CPL-bin-sum. These findings also confirm the observations of early reports that stallions exhibiting a heavy calibre, large frame, strong limbs, and bones with large articulations were more often affected by CPL [2,3,5,30]. In consequence, preferring those stallions in the decision for breeding would promote higher risk of CPL in their progeny [2,3,5,30]. In addition, we found that horses with longer lengths of the dorsal hoof walls may be genetically related with higher CPL scores, but not with a higher CPL prevalence. Shore D hardness seems to be negatively genetically correlated with the prevalence and severity of CPL. This may indicate that there could be a genetic relationship between hoof horn structure and CPL; this may support a relationship between an abnormal proliferation and differentiation of keratinocytes and CPL [13]. This finding may also explain the increasing prominence of chestnuts, ergots, and bulges in the fetlocks with increasing CPL scores [13,20]. Therefore, we propose that the pathogenesis of CPL may also be influenced by the disturbance of the proliferation and differentiation of keratinocytes, resulting in epidermal hyperplasia and an excessive production of keratin [13]. Thus, pathogenesis of CPL may be complicated by a further factor, which also has a genetic determination.

Skinfold thickness showed moderate positive additive genetic correlations of 0.440, 0.450, and 0.241 with CPL-bin-score, CPL-bin-sum, and the CPL score, respectively. Heritability of skinfold thickness and additive genetic correlation with CPL-bin-score agreed very well with the previous study across all German draught horse breeds and for South German draught horses [10,12]. On the other hand, the results for the Belgian draught horse were inconsistent between all horses and the > 3-year-old horses. In addition, a heritability estimate of 0.01 for skinfold thickness with a standard error of 0.02 can lead to uncertainty issues when estimating genetic correlations, as even small changes in the additive genetic variance have a large impact on the outcomes [22]. The genetic association between skinfold thickness and prevalence of CPL in German draught horses may also indicate that a general epidermal hyperplasia may drive dermal thickness with hyperkeratosis and the overproduction of keratin in the distal limbs.

Infestation with *Chorioptes* mites and bacterial colonisation are secondary complications in the distal limbs along with the progressing of CPL [9,10,12,13,14,21], but susceptibility to infestations with *Chorioptes* mites showed high heritabilities in German draught horse breeds [10,12] and moderate additive genetic correlations with CPL prevalence in South German draught horses [10,12]. Therefore, we may assume that significant additive genetic differences between horses in skin immunity and food supply and environmental conditions for *Chorioptes* mites, provided by skin scales, crusts, and excessive amounts of keratin, contribute to the increased susceptibility to these secondary complications with CPL [13,14,21].

In summary, the present study corroborated the significant contribution of genetics to the prevalence and expression of CPL lesions with different degrees of severity in Rhenish German. In addition, the impact of breeding for heavy calibre, large frame, strong limbs, and bones with large articulations became obvious as additional genetically disposing factors through the additive genetic correlations shown in this study.

## 5. Conclusions

The results of this study demonstrate high heritabilities with reasonable small standard errors for CPL scores using pedigree-based and genomic relationship matrices. Differentiation only between unaffected and affected horses revealed moderate heritabilities, suggesting that heritabilities increase with increasing severity of CPL lesions. The lower heritability for CPL prevalence may suggest that the genetic disposition for CPL is widespread in the Rhenish German population studied here and that the additive genetic differences between horse families are not as great as the differences in the severity of CPL lesions. We propose that breeding for lower CPL scores can exploit larger additive genetic differences among breeding animals. We recommend as a first step that a breeding programme should achieve a lower rate of CPL progression and thus contribute to less harmful lesions, particularly in older animals. A breeding programme aiming to reduce the prevalence in the shortest possible time may not be feasible due to the small population size and a very limited number of stallions being unaffected until more than 10 years of age. Further studies seem necessary for implementing an effective genomic breeding programme based on single-step BLUP approaches against CPL without negative effects on the genetic diversity in the Rhenish German population.

## Figures and Tables

**Table 1:** Heritability estimates (h^2^) with their standard errors (SEs) for scores of chronic progressive lymphedema (CPL) in Rhenish German draught horses including CPL scores per limb, maximum CPL scores across front limbs and hind limbs (CPL-max-front and CPL-max-hind), sum of scores across both front or both hind limbs (CPL-sum-front and CPL-sum-hind), overall score across all four limbs (CPL score), highest CPL score per horse (CPL-max), and sum of CPL scores across all four limbs (CPL-tot) using univariate linear and threshold animal models and pedigree-based relationship matrices.

Scores for CPL	Linear Model		Threshold Model	
	h^2^	SE	h^2^	SE
CPL score for front left	0.324	0.090	0.448	0.147
CPL score for front right	0.448	0.098	0.477	0.149
CPL score for hind left	0.482	0.062	0.637	0.133
CPL score for hind right	0.473	0.063	0.575	0.133
CPL-max-front	0.333	0.085	0.444	0.138
CPL-max-hind	0.480	0.085	0.606	0.133
CPL-sum-front	0.323	0.055	-	-
CPL-sum-hind	0.332	0.063	-	-
CPL score	0.482	0.105	0.595	0.131
CPL-max	0.490	0.097	0.587	0.131
CPL-tot	0.451	0.064	-	-

**Table 2:** Heritability estimates (h^2^) with their standard errors (SEs) for the overall score across all four limbs of chronic progressive lymphedema (CPL score) in Rhenish German draught horses restricted for at least 1, 2, 3, 4, 5, 6, 7, and 8 years of age using univariate linear and threshold animal models and pedigree-based relationship matrices.

CPL Score Restricted by Age	No. of Horses	Linear Model		Threshold Model	
		h^2^	SE	h^2^	SE
≥1 year of age	426	0.527	0.106	0.662	0.136
≥2 years of age	385	0.541	0.104	0.696	0.144
≥3 years of age	354	0.587	0.111	0.720	0.150
≥4 years of age	313	0.565	0.112	0.691	0.165
≥5 years of age	284	0.619	0.115	0.652	0.185
≥6 years of age	256	0.534	0.120	0.650	0.193
≥7 years of age	227	0.574	0.113	0.755	0.175
≥8 years of age	209	0.635	0.106	0.751	0.179

**Table 3:** Heritability estimates (h^2^) with their standard errors (SEs) for the overall score across all four limbs of chronic progressive lymphedema (CPL score) in Rhenish German draught horses using univariate linear and threshold animal models and censoring for trait values of the CPL score for horses below a minimum age and pedigree-based relationship matrices.

CPL Score with Censoring	Linear Model		Threshold Model	
	h^2^	SE	h^2^	SE
Censored for <1 year of age	0.501	0.107	0.662	0.136
Censored for <2 years of age	0.503	0.108	0.657	0.142
Censored for <3 years of age	0.586	0.111	0.666	0.145
Censored for <4 years of age	0.615	0.136	0.671	0.151
Censored for <5 years of age	0.616	0.141	0.645	0.167
Censored for <6 years of age	0.635	0.169	0.621	0.174
Censored for <7 years of age	0.669	0.155	0.719	0.171
Censored for <8 years of age	0.752	0.153	0.774	0.157
Censored for <9 years of age	0.732	0.150	0.788	0.168
Censored for <10 years of age	0.649	0.183	0.758	0.183
Censored for <11 years of age	0.696	0.184	0.769	0.183
Censored for <12 years of age	0.518	0.197	0.635	0.235
Censored for <13 years of age	0.392	0.192	0.553	0.246
Censored for <14 years of age	0.382	0.198	0.507	0.249
Censored for <15 years of age	0.330	0.189	0.440	0.237

**Table 4:** Heritability estimates (h^2^) with their standard errors (SEs) for body traits and their additive genetic (r_g_), residual (r_e_), and phenotypic (r_p_) correlations (including their SE) with the overall score across all four limbs of chronic progressive lymphedema (CPL) in Rhenish German draught horses using a linear multivariate animal model.

Body Trait	Heritability		Genetic		Residual		Phenotypic
	h^2^	SE	r_g_	SE	r_e_	SE	r_p_
Height at withers	0.696	0.082	0.338	0.106	0.154	0.126	0.245
Body length	0.303	0.081	0.166	0.163	−0.050	0.091	0.026
Chest circumference	0.308	0.083	0.144	0.159	0.068	0.089	0.094
Skinfold thickness	0.338	0.086	0.241	0.167	0.051	0.108	0.121
**Front left limb**							
Cannon bone circumference	0.358	0.077	0.617	0.105	0.201	0.105	0.359
Coronary band circumference	0.831	0.059	0.240	0.092	0.037	0.092	0.154
Shore D hardness of hoof horn	0.343	0.074	−0.314	0.135	−0.064	0.135	−0.159
Length of the dorsal wall	0.626	0.085	−0.165	0.114	0.325	0.114	0.066
Length of the heel wall	0.293	0.088	0.452	0.146	0.123	0.146	0.237
Front hoof angle	0.259	0.076	0.062	0.167	0.162	0.167	0.126
**Hind right limb**							
Cannon bone circumference	0.580	0.097	0.529	0.092	0.395	0.092	0.457
Coronary band circumference	0.589	0.076	0.097	0.102	0.221	0.102	0.156
Shore D hardness of hoof horn	0.302	0.076	−0.191	0.141	−0.088	0.141	−0.124
Length of the dorsal wall	0.603	0.079	0.050	0.113	0.173	0.113	0.108
Length of the heel wall	0.077	0.057	0.289	0.300	0.052	0.300	0.090
Front hoof angle	0.508	0.097	−0.263	0.128	0.300	0.128	0.037

**Table 5:** Heritability estimates (h^2^) with their standard errors (SEs) for scores of chronic progressive lymphedema (CPL) in Rhenish German draught horses including CPL scores per limb, maximum CPL scores across front limbs and hind limbs (CPL-max-front and CPL-max-hind), sum of scores across both front or both hind limbs (CPL-sum-front and CPL-sum-hind), overall score across all four limbs (CPL score), highest CPL score per horse (CPL-max), and sum of CPL scores across all four limbs (CPL-tot) using univariate threshold or linear animal models with genomic relationship matrices.

Scores for CPL	Threshold or Linear Model	
	h^2^	SE
CPL score for front left	0.431	0.134
CPL score for front right	0.513	0.143
CPL score for hind left	0.580	0.123
CPL score for hind right	0.562	0.122
CPL-max-front	0.484	0.132
CPL-max-hind	0.570	0.120
CPL-sum-front *	0.317	0.104
CPL-sum-hind *	0.443	0.104
CPL score	0.600	0.118
CPL-max	0.600	0.118
CPL-tot *	0.412	0.103

* Linear model.

**Table 6:** Heritability estimates (h^2^) with their standard errors (SEs) for the overall score across all four limbs of chronic progressive lymphedema (CPL score) in Rhenish German draught horses restricted for at least 1, 2, 3, 4, 5, 6, 7, and 8 years of age using univariate threshold animal models with genomic relationship matrices.

CPL Score Restricted By Age	Threshold Model	
	h^2^	SE
≥1 year of age	0.667	0.120
≥2 years of age	0.709	0.128
≥3 years of age	0.703	0.135
≥4 years of age	0.650	0.149
≥5 years of age	0.576	0.161
≥6 years of age	0.574	0.173
≥7 years of age	0.712	0.173
≥8 years of age	0.778	0.153

**Table 7:** Heritability estimates (h^2^) with their standard errors (SEs) for the overall score across all four limbs of chronic progressive lymphedema (CPL score) in Rhenish German draught horses using univariate threshold animal models with genomic relationship matrices and censoring for trait values of the CPL score for horses below a minimum age.

CPL Score with Censoring	Threshold Model	
	h^2^	SE
Censored for <1 year of age	0.667	0.120
Censored for <2 years of age	0.658	0.123
Censored for <3 years of age	0.648	0.128
Censored for <4 years of age	0.638	0.136
Censored for <5 years of age	0.586	0.145
Censored for <6 years of age	0.579	0.152
Censored for <7 years of age	0.719	0.159
Censored for <8 years of age	0.812	0.132
Censored for <9 years of age	0.765	0.155
Censored for <10 years of age	0.730	0.166
Censored for <11 years of age	0.749	0.166
Censored for <12 years of age	0.604	0.224
Censored for <13 years of age	0.476	0.228
Censored for <14 years of age	0.395	0.225
Censored for <15 years of age	0.345	0.214

**Table 8:** Heritability estimates (h^2^) with their standard errors (SEs) for the overall score across all four limbs as 0/1-trait (CPL-bin-score) and the sum of binary scores over all four limbs (CPL-bin-sum) of chronic progressive lymphedema in Rhenish German draught horses for all ages and restricted for at least 1, 2, 3, 4, 5, 6, 7, and 8 years of age using univariate linear animal models with pedigree-based relationship matrices and threshold animal models with pedigree-based and genomic relationship matrices.

Trait	Linear Model Pedigree		Threshold Model Pedigree		Threshold Model Genomic	
	h^2^	SE	h^2^	SE	h^2^	SE
CPL-bin-score	0.113	0.067	0.189	0.064	0.176	0.061
CPL-bin-sum	0.166	0.084	0.375	0.164	0.433	0.170
CPL-bin-score restricted by age						
≥1 year of age	0.099	0.063	0.192	0.066	0.181	0.064
≥2 years of age	0.119	0.073	0.199	0.065	0.188	0.067
≥3 years of age	0.170	0.088	0.202	0.060	0.190	0.069
≥4 years of age	0.152	0.077	0.199	0.057	0.188	0.071
≥5 years of age	0.150	0.094	0.190	0.056	0.178	0.073
≥6 years of age	0.116	0.076	0.175	0.056	0.165	0.075
≥7 years of age	0.137	0.089	0.168	0.056	0.159	0.075
≥8 years of age	0.249	0.122	0.164	0.056	0.155	0.077

**Table 9:** Heritability estimates (h^2^) with their standard errors (SEs) for the overall binary score across all four limbs of chronic progressive lymphedema (CPL-bin-score) in Rhenish German draught horses using univariate threshold animal models and censoring for trait values of the CPL score for horses below a minimum age with pedigree-based and genomic relationship matrices.

CPL-Bin-Score with Censoring	Threshold Model, Pedigree		Threshold Model, Genomic	
	h^2^	SE	h^2^	SE
Censored for <1 year of age	0.193	0.066	0.180	0.064
Censored for <2 years of age	0.200	0.066	0.189	0.067
Censored for <3 years of age	0.203	0.062	0.193	0.068
Censored for <4 years of age	0.202	0.058	0.192	0.071
Censored for <5 years of age	0.191	0.056	0.181	0.072
Censored for <6 years of age	0.177	0.055	0.167	0.073
Censored for <7 years of age	0.168	0.053	0.158	0.071
Censored for <8 years of age	0.168	0.053	0.158	0.071
Censored for <9 years of age	0.157	0.053	0.149	0.071
Censored for <10 years of age	0.153	0.052	0.145	0.071
Censored for <11 years of age	0.144	0.051	0.139	0.071
Censored for <12 years of age	0.144	0.051	0.139	0.071
Censored for <13 years of age	0.120	0.050	0.118	0.069
Censored for <14 years of age	0.113	0.048	0.111	0.068
Censored for <15 years of age	0.104	0.046	0.103	0.064

**Table 10:** Additive genetic (r_g_), residual (r_e_), and phenotypic (r_p_) correlations (including their SE) with the overall score across all four limbs of chronic progressive lymphedema as 0/1-trait (CPL-bin-score) in Rhenish German draught horses using a linear multivariate animal model.

Body Trait	Genetic		Residual		Phenotypic
	r_g_	SE	r_e_	SE	r_p_
Height at withers	0.409	0.286	0.166	0.125	0.217
Body length	0.304	0.245	0.085	0.099	0.131
Chest circumference	0.334	0.177	0.087	0.093	0.138
Skinfold thickness	0.440	0.207	0.050	0.097	0.139
**Front left limb**					
Cannon bone circumference	0.686	0.169	0.020	0.085	0.175
Coronary band circumference	0.203	0.121	0.233	0.275	0.142
Shore D hardness of hoof horn	−0.352	0.174	0.100	0.119	−0.006
Length of the dorsal wall	−0.130	0.169	−0.022	0.140	−0.049
Length of the heel wall	0.310	0.215	−0.106	0.123	−0.020
Front hoof angle	−0.027	0.196	0.062	0.110	0.042
**Hind right limb**					
Cannon bone circumference	0.634	0.152	0.021	0.099	0.206
Coronary band circumference	0.048	0.237	0.188	0.129	0.148
Shore D hardness of hoof horn	−0.258	0.256	0.094	0.116	0.032
Length of the dorsal wall	0.102	0.169	0.045	0.145	0.058
Length of the heel wall	0.145	0.466	−0.022	0.112	−0.004
Front hoof angle	−0.121	0.185	0.108	0.148	0.030

## Data Availability

Restrictions apply to the availability of these data. Data were obtained from German horse owners, breeders, and the Rhenish, Westphalian, Brandenburg-Anhalt, Thuringian, and Lower Saxony breeder associations and are available from the authors at a reasonable request and with the permission of the horse owners.

## Supplementary Materials

The following supporting information can be downloaded at: https://www.mdpi.com/article/10.3390/ani14081214/s1, Figure S1. Photographs of Rhenish German draught horses. Table S1. Distribution of horses by age in years and scores of chronic progressive lymphedema (CPL on a scale from 1–6) in Rhenish German draught horses. Table S2. Means and standard deviations for body and hoof traits by males (n = 80), geldings (n = 55), and females (n = 261) in Rhenish German draught horses aged >1 year. Table S3. Levels of fixed effects for farm-related variables with number of horses and means of the CPL score, CPL-bin-score, and CPL-bin-sum for each level. Table S4. Additive genetic (above the diagonal), residual (below the diagonal), and phenotypic (below the diagonal) correlations including their standard errors between CPL scores of the different limbs in Rhenish German draught horses using a linear multivariate animal model. Table S5. Additive genetic (above the diagonal), residual (below the diagonal), and phenotypic (below the diagonal) correlations including their standard errors between the sum of CPL scores of the front and hind limbs and the maximum values of CPL scores of the front and hind limbs and the overall CPL score in Rhenish German draught horses using a linear multivariate animal model. Table S6a. Additive genetic, residual, and phenotypic correlations between traits of the front and hind limb including the standard errors for additive genetic and residual correlations using a linear multitrait animal model. Table S6b. Additive genetic, residual, and phenotypic correlations between body size traits and hoof measures of the front limb including the standard errors for additive genetic and residual correlations using a linear multitrait animal model. Table S6c. Additive genetic, residual, and phenotypic correlations between body size traits and hoof measures of the hind limb including the standard errors for additive genetic and residual correlations using a linear multitrait animal model. Table S7a. Heritabilities (on the diagonal) and additive genetic, residual, and phenotypic correlations between CPL score and CPL-bin-scores as well as body traits including the standard errors for additive genetic and residual correlations using a linear multitrait animal model with a pedigree-based relationship matrix. Table S7b. Additive genetic, residual, and phenotypic correlations of CPL score and CPL-bin-scores and hoof traits of the front and hind limb including the standard errors for additive genetic and residual correlations using a linear multitrait animal model with a pedigree-based relationship matrix. Table S7c. Heritabilities (on the diagonal) and additive genetic and residual correlations between CPL score and CPL-bin-scores including the standard errors for additive genetic and residual correlations using a threshold multitrait animal model with a genomic relationship matrix. Table S8. Additive genetic, residual, and phenotypic correlations (including their SE) with the sum of dichotomous CPL scores over all four limbs (CPL-bin-sum) of chronic progressive lymphedema in Rhenish German draught horses using a linear multivariate animal model. Table S9. Heritabilities with their standard errors (SEs) for body traits and their additive genetic and residual correlations (including their SE) with the overall score across all four limbs of chronic progressive lymphedema (CPL score) in Rhenish German draught horses using a threshold-linear multivariate animal model with genomic relationship matrices. Table S10. Additive genetic and residual correlations (including their SE) with the sum of dichotomous CPL scores over all four limbs (CPL-bin-sum) of chronic progressive lymphedema in Rhenish German draught horses using linear-threshold bivariate animal models and genomic relationship matrices. Table S11. Additive genetic and residual correlations (including their SE) with the dichotomous CPL scores across all four limbs (CPL-bin-score) of chronic progressive lymphedema in Rhenish German draught horses using linear-threshold bivariate animal models and genomic relationship matrices.
