# Supplementary material for: The Estimation of Genetic Parameters for Chronic Progressive Lymphedema and Body Traits in the Rhenish German Draught Horse
Source: Animals (Basel). 2024 Apr 18;14(8):1214. doi: 10.3390/ani14081214 (PMC11047459; doi:10.3390/ani14081214)
Supplement: Supplementary file 1 [file animals-14-01214-s001.zip › Supplementary-Figure-S1-Tables-S1-S11.pdf]

Figure S1. Photographs of Rhenisch German draught horses.

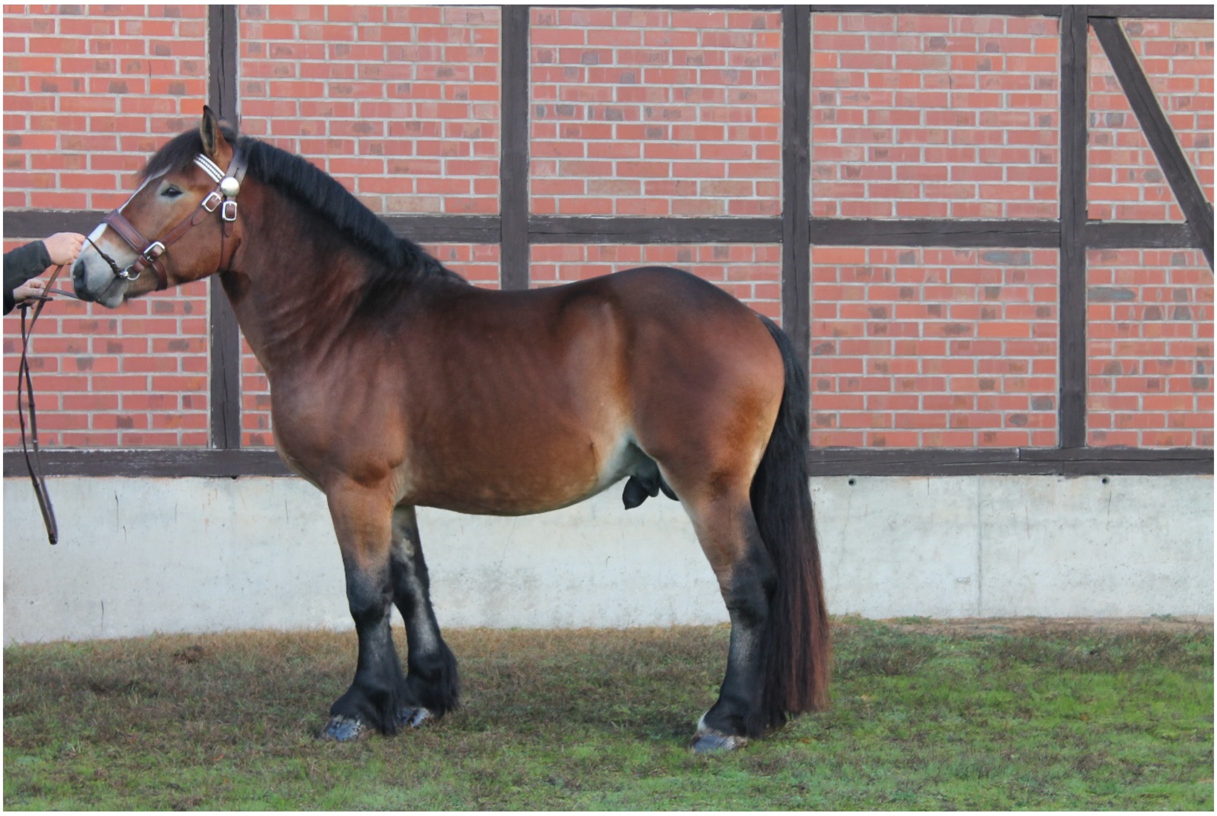

Young stallion at licensing

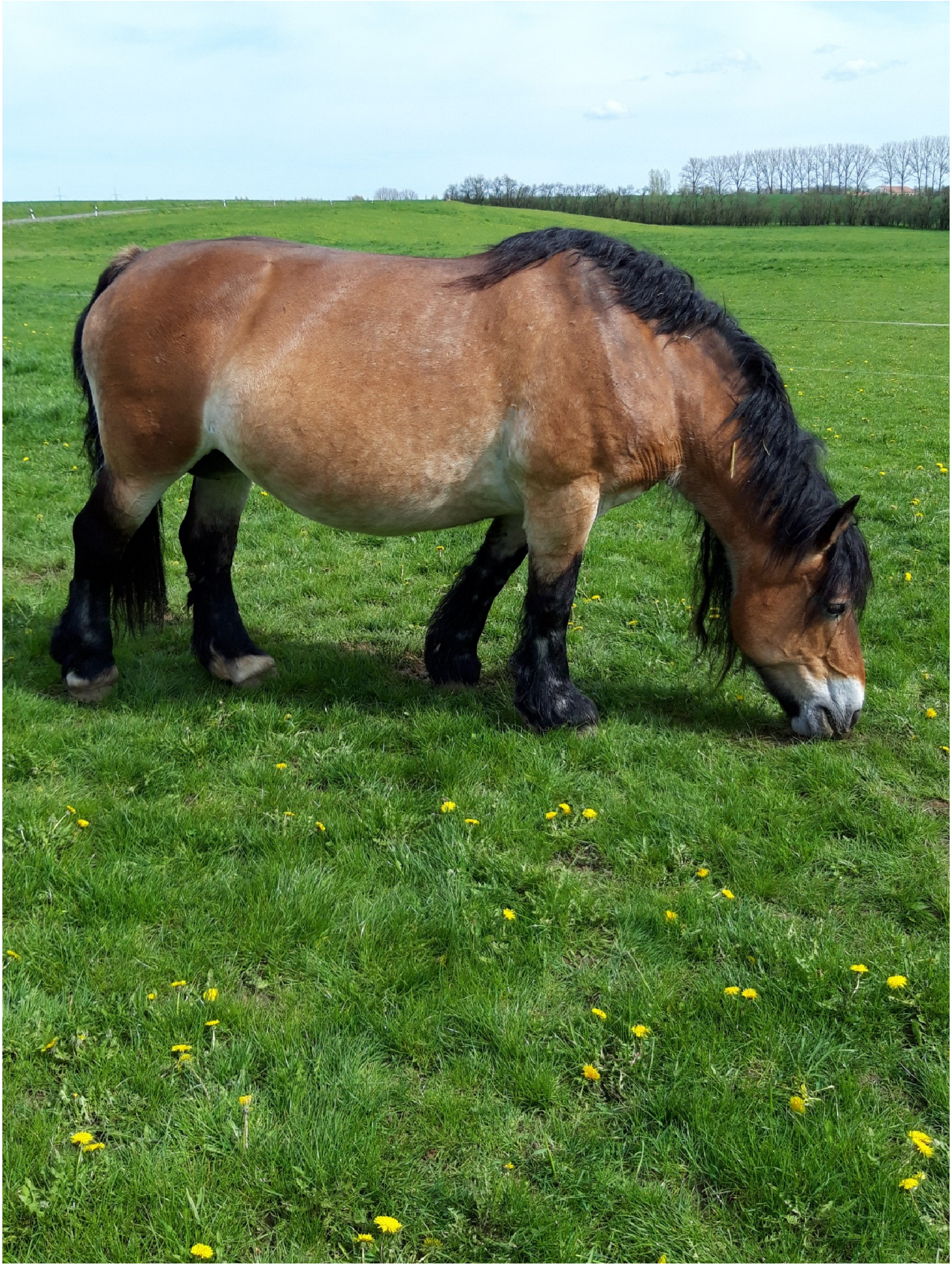

Broadmare at pasture

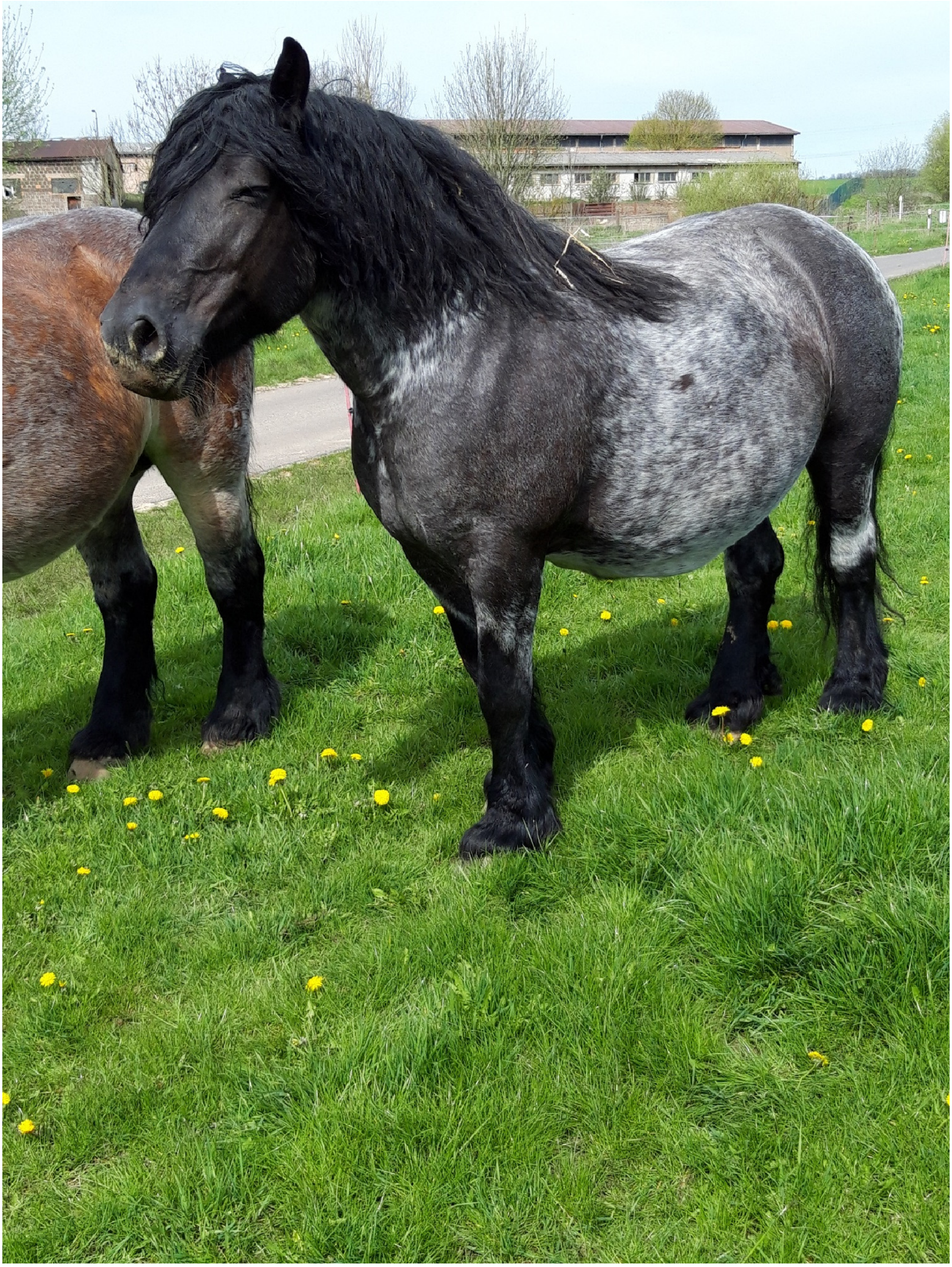

Broadmare at pasture

Table S1. Distribution of horses by age in years and scores of chronic progressive lymphedema (CPL on a scale from 1-6) in Rhenish German draught horses. Horses with a CPL-score of 1 are classified as unaffected and horses with a CPL-score of 2 and 6 with the mildest and most severe signs of CPL, respectively. The dichotomous CPL-score (CPL-bin-score, Cbin expressed as frequencies) differentiates between horses with a CPL-score of 1 (unaffected and 2-6 (affected) and CPL-bin-sum is the sum of the CPL-bin-score over all four limbs (Csum, Csum of 1 = no limb affected and Csum of 5 = all four limbs affected)

**Age (years)**	**No of horses per year and cumulative**		**CPL-score**							**Cbin**	**Csum**
			**1**	**2**	**3**	**4**	**5**	**6**	**Mean±SD**		
1	67	67	67	0	0	0	0	0	1.000 ± 0.000	0.00	1.00
2	41	108	32	5	3	1	0	0	1.341 ± 0.728	0.22	1.61
3	31	139	15	4	10	2	0	0	1.967 ± 1.048	0.52	2.74
4	41	180	19	5	13	4	0	0	2.049 ± 1.094	0.54	2.66
5	29	209	8	0	12	9	0	0	2.805 ± 1.222	0.72	3.59
6	28	237	8	1	10	9	0	0	2.714 ± 1.213	0.71	3.43
7	29	266	6	3	12	4	4	0	2.897 ± 1.291	0.79	3.76
8	18	284	2	2	5	5	4	0	3.389 ± 1.290	0.88	4.17
9	23	307	2	2	4	12	3	0	3.521 ± 1.123	0.91	4.26
10	26	333	2	3	5	10	5	1	3.615 ± 1.267	0.92	4.35
11	15	348	2	0	4	5	4	0	3.600 ± 1.298	0.87	4.27
12	21	369	0	3	4	10	3	1	3.762 ± 1.044	1.00	4.71
13	15	384	3	0	2	4	4	2	3.800 ± 1.699	0.80	4.27
14	15	399	1	0	3	6	5	0	3.933 ± 1.100	0.93	4.60
15	19	418	1	0	2	7	8	1	4.263 ± 1.098	0.95	4.63
16	12	430	1	0	5	3	3	0	3.583 ± 1.165	0.92	4.33
17	6	436	0	2	1	1	2	0	3.500 ± 1.378	1.00	4.00
18	15	451	0	0	1	7	7	0	4.400 ± 0.632	1.00	4.93
19	11	462	0	0	1	7	2	1	4.273 ± 0.786	1.00	4.91
20	9	471	0	0	1	6	2	0	4.111 ± 0.601	1.00	4.89
21	4	475	0	0	0	0	4	0	5.000 ± 0.000	1.00	5.00
22	7	482	1	0	1	5	0	0	3.428 ± 1.134	0.86	4.29
23	3	485	0	0	0	0	3	0	5.000 ± 0.000	1.00	5.00
24	2	487	0	1	0	1	0	0	-	1.00	3.50
25	2	489	0	0	1	0	1	0	-	1.00	4.50
26	1	490	0	0	0	1	0	0	-	1.00	4.00
27	1	491	0	0	0	1	0	0	-	1.00	5.00
28	1	492	0	0	0	0	1	0	-	1.00	5.00
34	1	493	0	0	1	0	0	0	-	1.00	5.00
Total (n)	493	493	170	31	101	120	65	6	2.791 ± 1.511	0.66 ± 0.48	3.35 ± 1.81
Percentage		100	34.5	6.3	20.5	24.3	13.2	1.2			

Table S2. Means and standard deviations for body and hoof traits by males (n=80), geldings (n=55) and females (n=261) in Rhenish German draught horses aged >1 year.

Variate	Male	Gelding	Female
Height at withers (cm)	162.21 ± 6.20	164.35 ± 6.05	160.54 ± 5.52
Body length (cm)	171.74 ± 8.51	176.42 ± 9.21	177.75 ± 8.63
Chest circumference (cm)	202.54 ± 10.53	214.06 ± 9.98	213.61 ± 13.22
Skinfold thickness at the neck region (mm)	7.16 ± 1.66	6.47 ± 0.95	5.50 ± 1.12
**Front left limb**			
Cannon bone circumference (cm)	27.41 ± 3.57	27.07 ± 1.88	25.50 ± 2.86
Circumference coronary band (cm)	49.92 ± 2.91	50.50 ± 4.98	48.38 ± 4.62
Shore D hardness of hoof horn	59.33 ± 11.07	64.10 ± 10.26	62.82 ± 9.95
Length of the dorsal wall (cm)	10.80 ± 1.29	10.65 ± 1.17	10.38 ± 1.05
Length of the heel wall cm)	7.37 ± 1.92	7.46 ± 1.81	7.02 ± 1.80
Front hoof angle (°)	52.74 ± 4.58	52.76 ± 6.00	51.39 ± 5.67
**Hind right limb**			
Circumference of cannon bone (cm)	30.96 ± 3.45	30.53 ± 2.16	28.66 ± 2.27
Circumference coronary band (cm)	49.25 ± 3.96	49.55 ± 4.93	48.16 ± 3.76
Shore D hardness of hoof horn	57.75 ± 12.62	65.74 ± 11.68	63.92 ± 10.66
Length of the dorsal wall (cm)	10.18 ± 1.05	10.48 ± 1.25	10.09 ± 1.02
Length of the heel wall (cm)	6.83 ± 1.61	7.16 ± 1.51	6.93 ± 5.00
Front hoof angle (°)	55.09 ± 5.38	53.81 ± 4.52	52.12 ± 5.92

Definitions:

Height at withers: distance between the highest point of the withers and the ground surface

Body length: distance between *tuberculum majus ossis humeri* and *tuber* ischiadicum (maximal oblique trunk length)

Chest circumference: circumference of the *thorax* behind the shoulders

Cannon bone circumference: metacarpal circumference or metatarsal circumference at the smallest diameter

Circumference coronary band: Circumference at the top of the hoof wall

Length of the dorsal wall: Length of dorsal border from horn junction at coronary band to apex of toe

Length of the heel wall: length of the heel wall at the angle of the bar

Front hoof angle: Angle of dorsal border to weight-bearing surface of the hoof wall

Table S3. Levels of fixed effects for farm-related variables with number of horses and means of the CPL-score, CPL-bin-score and CPL-bin-sum for each level.

Effect	Level and no of level		No of horses	CPL-score	CPL-bin-score	CPL-bin-sum
OUTS						
	Paddock with open barn	1	34	4.09	0.97	4.70
	Pasture	2	357	2.85	0.68	3.44
	Pasture and paddock	3	92	2.01	0.43	2.40
	Unknown	4	10	3.30	0.90	4.40
BED						
	Barley straw	1	57	3.67	0.86	4.14
	Rye straw	2	16	3.12	0.75	3.31
	Wheat straw	3	127	2.82	0.69	3.46
	Wood shavings	4	12	4.00	1.00	5.00
	Barley + wheat straw	5	167	2.50	0.56	2.96
	Rye + wheat straw	6	55	1.84	0.42	2.36
	Any straw	7	28	3.57	0.86	4.32
	Unknown	8	31	2.97	0.74	3.87
CLEAN						
	1-7 days	1	254	2.79	0.65	3.35
	2-4 weeks	2	145	2.59	0.61	3.12
	1-2 months	3	63	3.14	0.73	3.67
	>2 months	4	31	3.03	0.74	3.84
ROUW						
	Hay	1	198	2.75	0.66	3.35
	Hay + straw	2	89	3.11	0.72	3.61
	Hay + silage or haysilage	3	153	2.46	0.54	2.92
	Haysilage	4	39	3.41	0.85	4.21
	Unknown	5	14	3.21	0.86	4.29
CONW						
	Concentrates	1	377	2.90	0.68	3.46
	No concentrates	2	116	2.44	0.58	3.03
HOFT						
	4–8 weeks	1	147	3.56	0.86	4.24
	9–12 weeks	2	105	3.08	0.75	3.65
	13–16 weeks	3	85	2.58	0.65	3.22
	>16 weeks	4	26	2.23	0.58	2.81
	Unknown	5	130	1.94	0.37	2.32

Abbreviations: OUTS = Outdoor facilities for horses in summer, BED = Bedding type, CLEAN = Time interval for cleaning out the stable, ROUW = Type of roughage fed in winter months, CONW = Type of concentrate fed in winter months, HOFT = Length of hoof trimming intervals.

Table S4. Additive genetic (above the diagonal), residual (below the diagonal) and phenotypic (r_P) correlations (below the diagonal) including their standard errors between CPL-scores of the different limbs in Rhenish German draught horses using a linear multivariate animal model.

CPL-Scores	Front left		Front right		Hind left		Hind right	Overall CPL-score	
		r_P		r_P		r_P			r_P
Front left	-		0.992 ± 0.147		0.942 ± 0.199		0.919 ± 0.114	0.966 ± 0.034	0.791
Front right	0.752 ± 0.046	0.816	-		0.956 ± 0.140		0.911 ± 0.105	0.910 ± 0.051	0.832
Hind left	0.547 ± 0.111	0.666	0.560 ± 0.166	0.736	-		0.979 ± 0.043	0.959 ± 0.027	0.842
Hind right	0.529 ± 0.151	0.647	0.640 ± 0.105	0.832	0.731 ± 0.185	0.842	-	0.935 ± 0.031	0.863
Overall CPL-score	0.729 ± 0.051		0.796 ± 0.048		0.742 ± 0.033		0.802 ± 0.031	-	-

Table S5. Additive genetic (above the diagonal), residual (below the diagonal) and phenotypic (r_P) correlations (below the diagonal) including their standard errors between the sum of CPL-scores of the front and hind limbs and the maximum values of CPL-scores of the front and hind limbs and the overall CPL-score in Rhenish German draught horses using a linear multivariate animal model.

CPL-Scores	Front sum		Hind sum		Front max		Hind max	Overall CPL-score	
		r_P		r_P		r_P			r_P
Front sum	-		0.902 ± 0.059		0.986 ± 0.015		0.902 ± 0.059	0.954 ± 0.045	0.855
Hind sum	0.898 ± 0.065	0.752	-		0.942 ± 0.047		0.995 ± 0.005	0.939 ± 0.031	0.886
Front max	0.949 ± 0.010	0.959	0.627 ± 0.062	0.732	-		0.948 ± 0.049	0.932 ± 0.062	0.861
Hind max	0.627 ± 0.066	0.735	0.939 ± 0.013	0.967	0.586 ± 0.068	0.723	-	0.986 ± 0.019	0.901
Overall CPL-score	0.803 ± 0.042	0.856	0.837 ± 0.038	0.887	0.807 ± 0.067	0.861	0.796 ± 0.019	-	-

Table S8. Additive genetic, residual and phenotypic correlations (including their SE) with the sum of dichotomous CPL-scores over all four limbs (CPL-bin-sum) of chronic progressive lymphedema in Rhenish German draught horses using a linear multivariate animal model.

Body trait	Genetic		Residual		Phenotypic
	r_g	SE	r_e	SE	r_p
Height at withers	0.492	0.120	0.184	0.104	0.239
Body length	0.367	0.170	0.039	0.076	0.104
Chest circumference	0.105	0.161	0.103	0.074	0.101
Skinfold thickness	0.450	0.123	0.032	0.086	0.117
**Front left limb**					
Cannon bone circumference	0.695	0.191	0.135	0.076	0.250
Circumference coronary band	0.070	0.136	0.196	0.235	0.082
Shore D hardness of hoof horn	-0.358	0.115	0.031	0.087	-0.055
Length of the dorsal wall	-0.002	0.169	0.100	0.108	0.052
Length of the heel wall	0.300	0.194	0.040	0.088	0.093
Front hoof angle	-0.093	0.163	0.027	0.076	0.039
**Hind right limb**					
Cannon bone circumference	0.738	0.108	0.160	0.091	0.297
Circumference coronary band	0.031	0.175	0.251	0.103	0.176
Shore D hardness of hoof horn	-0.373	0.208	0.024	0.083	-0.039
Length of the dorsal wall	-0.067	0.159	0.194	0.111	0.101
Length of the heel wall	0.277	0.343	0.034	0.075	0.056
Front hoof angle	-0.050	0.129	0.117	0.112	0.053

Table S9. Heritabilities with their standard errors (SE) for body traits and their additive genetic and residual correlations (including their SE) with the overall score across all four limbs of chronic progressive lymphedema (CPL-score) in Rhenish German draught horses using a threshold-linear multivariate animal model with genomic relationship matrices.

Body trait	Heritability		Genetic		Residual	
	h^2	SE	r_g	SE	r_e	SE
Height at withers	0.557	0.118	0.555	0.201	-0.005	0.238
Body length	0.175	0.122	0.195	0.392	-0.126	0.160
Chest circumference	0.230	0.112	0.693	0.266	-0.223	0.184
Skinfold thickness	0.343	0.120	0.517	0.202	0.011	0.162
**Front left limb**						
Cannon bone circumference	0.351	0.085	0.825	0.116	0.147	0.126
Circumference coronary band	0.779	0.143	0.488	0.178	-0.549	0.315
Shore D hardness of hoof horn	0.358	0.151	-0.517	0.316	0.030	0.213
Length of the dorsal wall	0.586	0.130	-0.007	0.188	0.227	0.229
Length of the heel wall	0.334	0.115	0.552	0.252	0.083	0.158
Front hoof angle	0.153	0.110	0.283	0.488	0.177	0.178
**Hind right limb**						
Cannon bone circumference	0.548	0.099	0.671	0.141	0.484	0.149
Circumference coronary band	0.369	0.127	0.143	0.238	0.172	0.181
Shore D hardness of hoof horn	0.234	0.147	-0.304	0.321	-0.167	0.165
Length of the dorsal wall	0.602	0.117	0.006	0.203	0.182	0.222
Length of the heel wall	0.092	0.084	0.257	0.561	0.040	0.201
Front hoof angle	0.549	0.161	-0.269	0.290	0.422	0.229

Table S10. Additive genetic and residual correlations (including their SE) with the sum of dichotomous CPL-scores over all four limbs (CPL-bin-sum) of chronic progressive lymphedema in Rhenish German draught horses using linear-threshold bivariate animal models and genomic relationship matrices.

Body trait	Genetic		Residual	
	r_g	SE	r_e	SE
Height at withers	0.661	0.218	0.128	0.209
Body length	0.301	0.517	-0.039	0.162
Chest circumference	0.693	0.290	-0.106	0.199
Skinfold thickness	0.609	0.226	0.116	0.195
**Front left limb**				
Cannon bone circumference	0.729	0.173	0.680	0.132
Circumference coronary band	0.198	0.114	0.135	0.105
Shore D hardness of hoof horn	-0.310	0.137	-0.036	0.081
Length of the dorsal wall	0.047	0.134	0.222	0.075
Length of the heel wall	0.563	0.264	-0.052	0.083
Front hoof angle	0.457	0.152	-0.208	0.086
**Hind right limb**				
Cannon bone circumference	0.687	0.200	0.538	0.168
Circumference coronary band	-0.287	0.326	0.598	0.237
Shore D hardness of hoof horn	-0.305	0.151	-0.073	0.199
Length of the dorsal wall	-0.306	0.311	0.494	0.253
Length of the heel wall	0.481	0.587	0.220	0.257
Front hoof angle	-0.230	0.392	0.270	0.278

Table S11. Additive genetic and residual correlations (including their SE) with the dichotomous CPL-score across all four limbs (CPL-bin-score) of chronic progressive lymphedema in Rhenish German draught horses using a linear-threshold bivariate animal models and genomic relationship matrices.

Body trait	Genetic		Residual	
	r_g	SE	r_e	SE
Height at withers	0.793	0.240	0.208	0.160
Body length	0.054	0.634	0.189	0.133
Chest circumference	0.556	0.435	0.171	0.133
Skinfold thickness	0.784	0.285	0.177	0.146
**Front left limb**				
Cannon bone circumference	0.868	0.202	0.264	0.141
Circumference coronary band	0.456	0.520	0.019	0.290
Shore D hardness of hoof horn	-0.446	0.518	0.020	0.165
Length of the dorsal wall	0.137	0.541	0.044	0.178
Length of the heel wall	0.449	0.536	0.090	0.168
Front hoof angle	0.062	0.688	0.096	0.166
**Hind right limb**				
Cannon bone circumference	0.867	0.154	0.316	0.145
Circumference coronary band	-0.404	0.536	0.366	0.178
Shore D hardness of hoof horn	-0.235	0.665	-0.055	0.170
Length of the dorsal wall	-0.487	0.427	0.331	0.213
Length of the heel wall	-0.151	0.679	0.080	0.128
Front hoof angle	-0.196	0.633	0.170	0.214
